# Percutaneous Transhepatic Sphincterotome–Guided Management of Post–Living Donor Liver Transplant Biliary Anastomotic Stricture: An Innovative Approach

**DOI:** 10.14309/crj.0000000000001288

**Published:** 2024-03-22

**Authors:** Usman Iqbal Aujla, Imran Ali Syed, Ahmad Karim Malik, Muhammad Ramzan, Abdullah Saeed

**Affiliations:** 1 Gastroenterology and Hepatology Department, Pakistan Kidney and Liver Institute & Research Center, DHA Phase VI, Lahore, Pakistan; 2 Department of Radiology, Pakistan Kidney and Liver Institute & Research Center, DHA Phase VI, Lahore, Pakistan

**Keywords:** liver transplant, biliary complications, anastomotic stricture, sphincterotome, endoscopic retrograde cholangiopancreatography, intraductal cholangioscopy

## Abstract

Post–liver transplantation biliary complications remain a serious concern and are associated with reduced patient and graft survival. Among various biliary complications, anastomotic stricture (AS) is the most frequent and challenging one. The frequency of AS after living donor liver transplantation (LDLT) is higher as compared to deceased donor liver transplantation. The management involves endoscopic retrograde cholangiopancreatography and/or percutaneous transhepatic biliary drainage, but refractory cases necessitate surgical revision. We present a case of complex biliary AS in a 63-year-old man after LDLT. The conventional approaches including endoscopic retrograde cholangiopancreatography, percutaneous transhepatic cholangiography, and cholangioscope-guided interventions remained unsuccessful. An innovative approach using a wire-guided sphincterotome through percutaneous transhepatic route successfully managed the complex post-LDLT AS. This is perhaps the first reported case of novel utilization of sphincterotome through transhepatic route for the management of AS in LDLT, averting major surgical interventions with related morbidity and mortality.

## INTRODUCTION

Biliary complications remain the “Achilles heel” of liver transplantation (LT) and may affect graft function and the recipient's survival. These include biliary strictures (anastomotic and nonanastomotic), bile leaks, and stones. Approximately 50% of all the biliary complications occur at the site of biliary anastomosis with an overall frequency of 15%–30% after living donor liver transplantation (LDLT) and 10%–15% after deceased donor liver transplant (DDLT).^1^ Biliary anastomotic strictures (AS) represent the most frequent biliary complication and occur in 9%–12% of recipients after DDLT and 12%–30% after LDLT.^2^

A comprehensive retrospective analysis of 8,304 liver transplants revealed that recipients with early anastomotic biliary complications, compared with those without such complications, experienced reduced 5-year patient survival (76.9% vs 83.3%) and graft survival (75.1% vs 84.5%).^3^

Various risk factors for AS have been identified and include recipient factors (older age and high model for end-stage liver disease score), graft factors (advanced donor age >50 years, right lateral sector graft, smaller bile duct diameter, and bile duct multiplicity), operative factors (longer warm or cold ischemia time and hepaticojejunostomy) and various post-transplant factors (cytomegalovirus infection, preceding bile leakage, and hepatic artery thrombosis).^4^ Clinical manifestations include fever, jaundice, and right hypochondrial pain along with abnormal liver function tests. Ultrasound is the primary modality used to detect AS, with a reported sensitivity of 30%–70%.^5^ Magnetic resonance cholangiopancreatography (MRCP) remains the preferred noninvasive radiological modality to evaluate AS and serves as a roadmap to guide treatment interventions. The reported sensitivity and specificity of MRCP approaches 97% and 98%, respectively, in accurate detection of biliary strictures.^6^ Primary treatment modalities include endoscopic retrograde cholangiopancreatography (ERCP) and/or percutaneous transhepatic cholangiography (PTC), whereas surgical revision is reserved for intractable strictures.^7^ In this article, we present a case of post-LDLT biliary AS with previous unsuccessful management with ERCP, PTC, and intraductal cholangioscopy. It was treated successfully through novel utilization of wire-guided sphincterotome through percutaneous transhepatic route, consequently averting the need for a major surgery with related morbidity and mortality.

## CASE REPORT

A 63-year-old man underwent LDLT because of hepatitis C virus–related decompensated liver disease complicated with hepatocellular carcinoma. He received a right lobe graft, and biliary anastomosis was constructed between the recipient's common hepatic duct and the graft's right hepatic duct.

**Figure 1.**
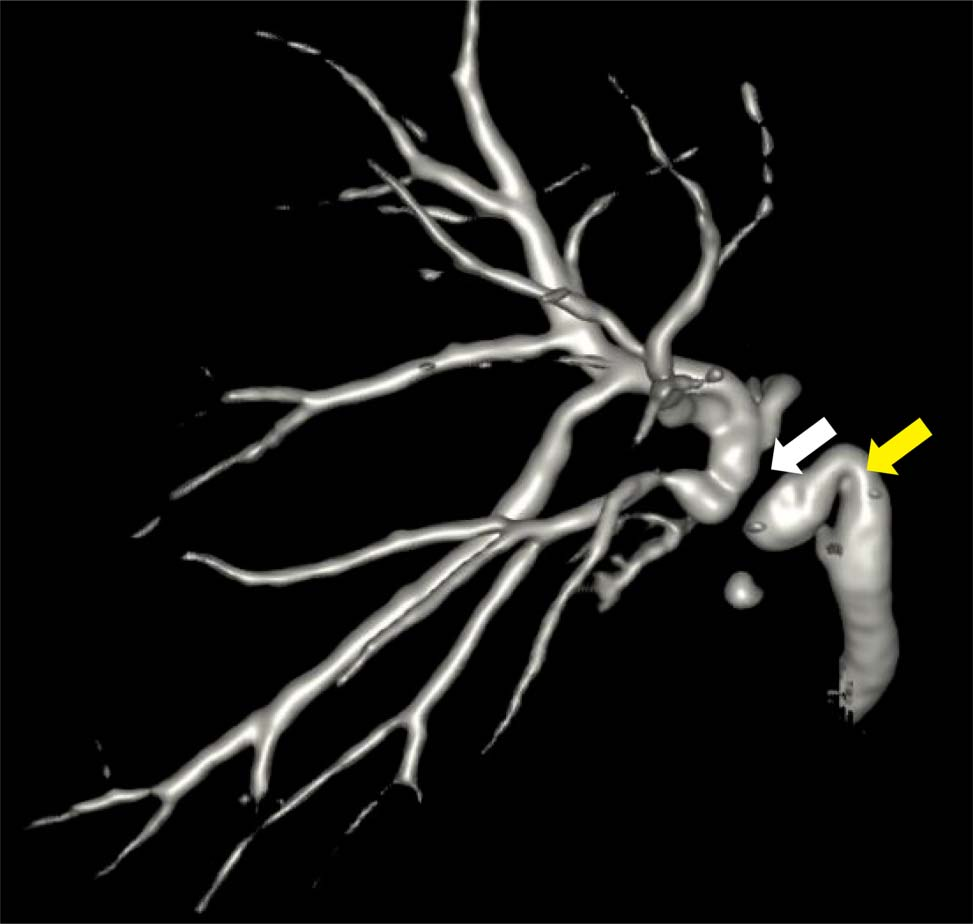
Magnetic resonance cholangiography showing a tight biliary anastomotic stricture (white arrow) and a sharply angulated native bile duct (yellow arrow).

**Figure 2.**
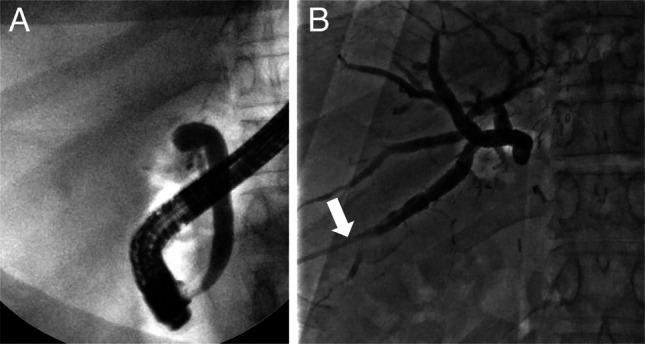
(A) ERCP showing angulated bile duct with a complete stenosis of anastomotic site not allowing for passage of contrast. (B) Percutaneous transhepatic cholangiography with an external drain (white arrow). ERCP, endoscopic retrograde cholangiography.

He presented 2 months after LDLT with a 1-week history of jaundice, pruritis, and dark-colored urine. He denied any history of fever, rigors, and weight loss. Post–liver transplant immunosuppression included tacrolimus 1.5 mg twice a day and prednisolone 5 mg once daily. His liver function tests revealed a total bilirubin of 8.9 mg/dL (direct bilirubin 7.2 mg/dL), alanine aminotransferase 840 U/L, aspartate aminotransferase 451 U/L, alkaline phosphatase 1382 U/L, and γ-glutamyl transpeptidase 1,263 U/L. His serum tacrolimus level was 9.2 ng/mL. Hepatitis C virus RNA and cytomegalovirus DNA remained undetected on polymerase chain reaction tests. MRCP was performed, which revealed a biliary AS with resultant upstream intrahepatic biliary dilatation (Figure 1). Subsequently, ERCP was attempted to manage the stricture, which remained unsuccessful because of very tight AS (Figure 2). Intraductal cholangioscope–guided intervention deemed technically unfeasible because of sharp angulation at the biliary anastomotic site. The patient was referred to interventional radiology for percutaneous management of stricture. However, stricture could not be traversed through the transhepatic route, and an external biliary drain was placed to relieve jaundice (Figure 2). Two weeks later, a repeat attempt to internalize the percutaneous drain remained unsuccessful. Hence, it was decided to traverse the AS with transhepatic cholangioscope-guided interventions. However, extremely sharp angulation at the anastomotic site and twisted duct of the graft limited the approximation of the cholangioscope to the anastomotic site.

**Figure 3.**
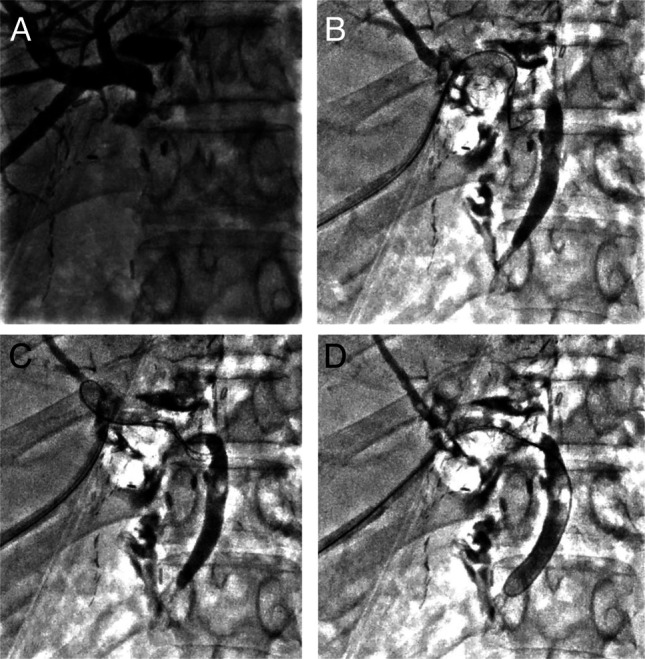
(A) Percutaneous transhepatic cholangiography showing anastomotic stricture. (B) Anastomotic stricture successfully traversed by manual introduction of sphincterotome via the percutaneous route. (C and D) The guide wire is adeptly threaded through the tight and angled stricture, successfully reaching the common bile duct through a series of precise 360° rotating maneuvers manually performed with the sphincterotome.

To overcome the anatomical challenge, an ERCP sphincterotome was advanced over the PTC sheath into the biliary system. By exerting traction on the cutting wire, the sphincterotome's tip allowed for flexible adjustment of the angulation to 116°. This maneuver brought the sphincterotome tip in close proximity to the stricture. Under fluoroscopic guidance, complex 360° rotating maneuvers of the sphincterotome facilitated the smooth passage of single-use straight tip (0.035-inch) guidewire across the narrow stricture (Figure 3).

**Figure 4.**
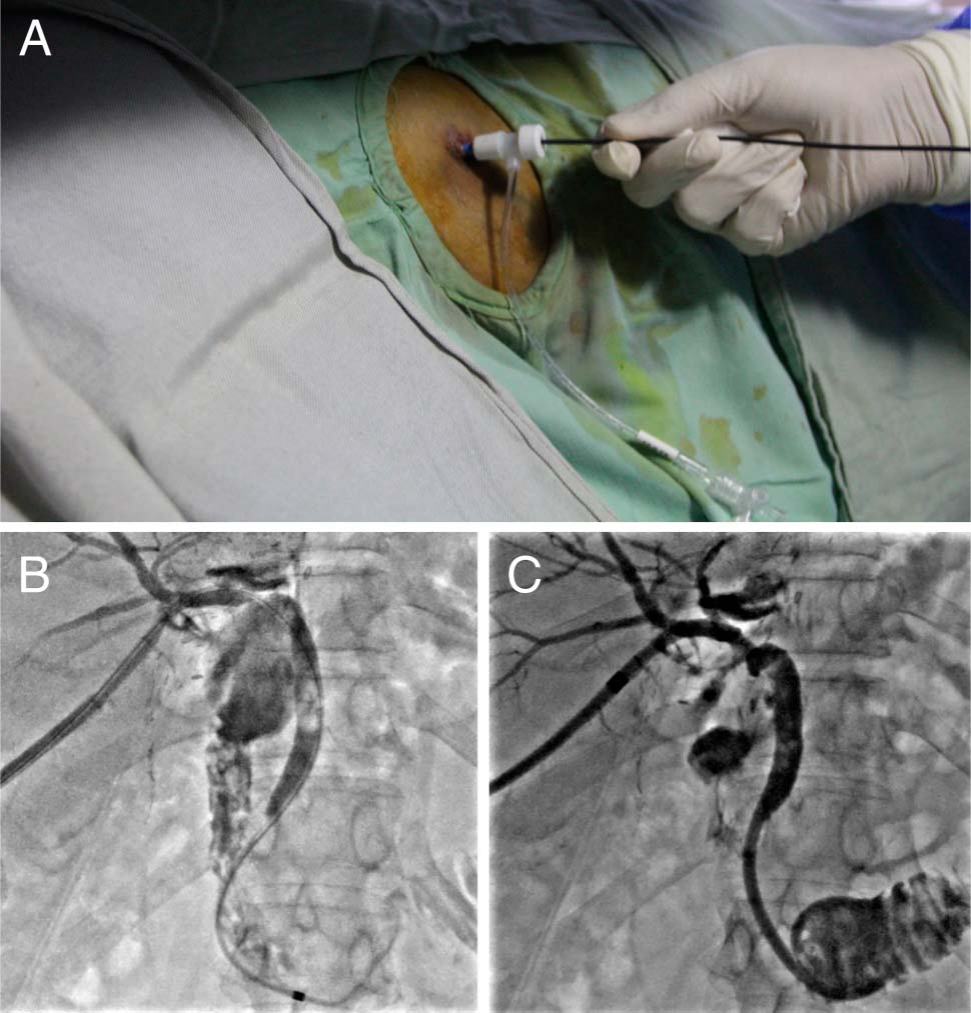
(A) Introduction of 7 French biliary dilation catheter through percutaneous transhepatic route to dilate the stricture. (B) Cholangiogram showing stricture dilatation being performed using a 7 French biliary dilation catheter. (C) Placement of percutaneous transhepatic biliary drainage catheter.

**Figure 5.**
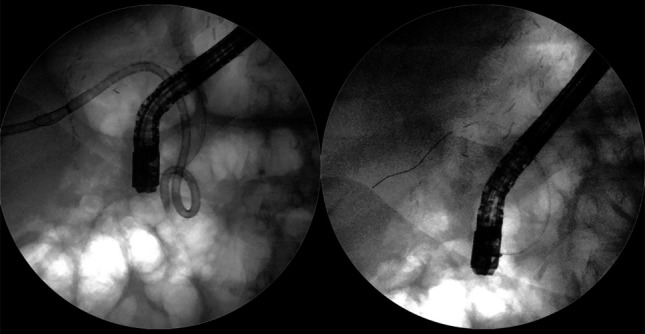
Endoscopic retrograde cholangiography showing transpapillary placement of guidewire along PTBD catheter followed by removal of the PTBD catheter. PTBD, percutaneous transhepatic biliary drainage.

**Figure 6.**
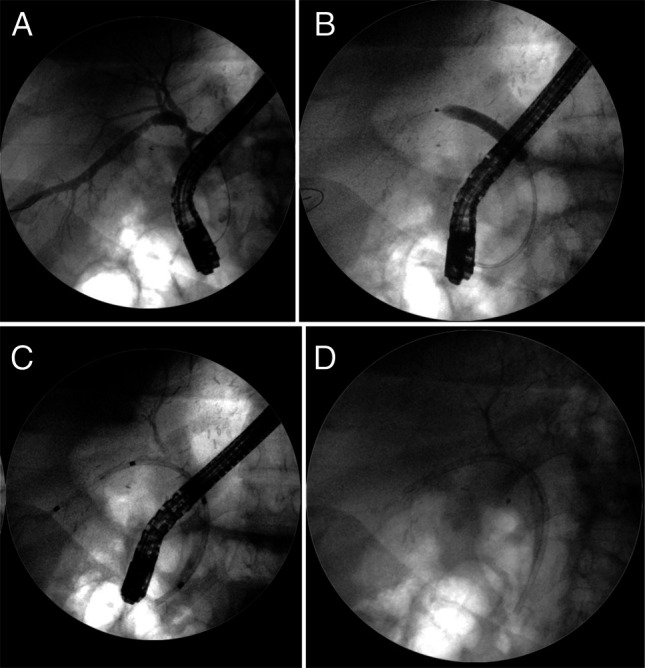
(A) Occlusion cholangiogram showing biliary anastomotic stricture. (B) Balloon dilatation being performed at stricture site. (C & D) Single plastic biliary stent deployed successfully across the stricture over the guide catheter.

The stricture was dilated with a 7 French biliary dilatation catheter followed by successful placement of internal-external biliary drain (Figure 4). There were no procedure-related complications. The external-internal drain was replaced with a single plastic biliary stent on subsequent ERCP (Figures 5 and 6). Subsequently, patient's LFTs completely normalized with no ongoing biliary symptoms.

## DISCUSSION

ASs remain the most frequent and debilitating biliary complication after LDLT with a reported prevalence of around 6.6% at 1 year and 12.3% at 10 years.^8^ A marked paradigm shift in the management strategies of AS has been observed, transitioning from predominantly surgical management to primarily endoscopic (ERCP) and/or percutaneous approaches.^4^ The sustained patency rates for AS after ERCP falls around 80%–90% in DDLT, whereas slightly lower rates (60%–84%) are reported in LDLT.^9^ Although percutaneous transhepatic biliary drainage demonstrates comparable success rates with ERCP, it is associated with an increased risk of complications including infections, drain dislodgement, patient discomfort, and higher recurrence rates.^9–12^ The primary endoscopic management techniques include stricture dilatation and/or placement of biliary stents. The risk of treatment failure and recurrence rates is significantly elevated when only balloon dilatation is performed or a single stent is placed, in contrast to the utilization of multiple plastic stents.^9,12^ A meta-analysis evaluating the efficacy of multiple plastic stents revealed stricture resolution rates ranging from 94% to 100%.^13^ Refractory AS poses a management dilemma, but innovations in ERCP have shown improved outcomes with magnetic compression anastomosis and cholangioscopy-guided steroid injections. Overall success rate of magnetic compression anastomosis in treating refractory AS is reported to be around 88%.^12,14^ Failure of the endoscopic and percutaneous techniques renders the patient for a surgical revision, which includes reanastomosis, bilioenterostomy, and rarely retransplantation. To the best of our knowledge, this is the first case report of transhepatic utilization of ERCP sphincterotome to traverse the AS and provision of subsequent stricture management in the setting of LDLT.

This case highlights the novel utilization of sphincterotome through the transhepatic route to negotiate the AS with the successful placement of biliary stents. This innovative technique could be considered when traditional endoscopic and percutaneous approaches remain unsuccessful, averting major surgical interventions with related implications.

## DISCLOSURES

Author contributions: UI Aujla: wrote the manuscript and critical analysis, approved the final version, and is the article guarantor. IA Syed: wrote the manuscript and reviewed the literature. AK Malik: provided endoscopic images and described the findings. M. Ramzan and A. Saeed: reviewed the literature, described radiological images, and revised the manuscript.

Financial disclosure: None to report.

Informed consent was obtained for this case report.
